# Altered specificity of single-chain antibody fragments bound to pandemic H1N1-2009 influenza virus after conversion of the phage-bound to the soluble form

**DOI:** 10.1186/1756-0500-5-483

**Published:** 2012-09-04

**Authors:** Yoshihiro Kaku, Akira Noguchi, Akiko Okutani, Satoshi Inoue, Kiyoshi Tanabayashi, Yoshie Yamamoto, Akitoyo Hotta, Michio Suzuki, Naoko Sugiura, Akio Yamada

**Affiliations:** 1 Department of Veterinary Science, National Institute of Infectious Diseases, Shinjuku, Tokyo, 162-8640, Japan

**Keywords:** Influenza, Pandemic, Diagnosis, Single-chain variable fragment (scFv), Altered specificity

## Abstract

**Background:**

In 2009, a novel influenza A/H1N1 virus (H1N1pdm) quickly spread worldwide and co-circulated with then-existing seasonal H1N1 virus (sH1N1). Distinguishing between these 2 viruses was necessary to better characterize the epidemiological properties of the emergent virus, including transmission patterns, pathogenesis, and anti-influenza drug resistance. This situation prompted us to develop a point-of-care virus differentiation system before entering the 2009–2010 influenza season. Aiming to establish H1N1pdm-specific detection tools rapidly, we employed phage display libraries to select H1N1pdm-specific single-chain variable fragments (scFvs).

**Findings:**

Human single-fold scFv libraries (Tomlinson I + J) underwent selection for the ability to bind H1N1pdm virus particles. Three rounds of panning brought 1152 phage-bound scFvs, of which 58 clones reacted with H1N1pdm specifically or preferentially over sH1N1 in an enzyme-linked immunosorbent assay (ELISA). After conversion of the scFvs to soluble form, 7 clones demonstrating high/stable expression were finally obtained. However, all the soluble scFvs except No. 29 were found to have lost their specificity/preference for H1N1pdm in ELISA. The specificity/preference of No. 29 was also confirmed by immunofluorescence assay and immunoprecipitation, and the viral nucleoprotein was identified by ELISA as its target protein. The change in specificity associated with scFv conversion from phage-bound to soluble form could be due to loss of phage scaffold pIII protein, which likely provides structural support for the scFv antigen-binding site. It is also possible that the similar antigenic properties of H1N1pdm and sH1N1 led to the observed alterations in scFv specificity.

**Discussion:**

Using a phage display library, we obtained 7 soluble scFv clones reactive against H1N1pdm; however, only 1 showed specificity/preference toward H1N1pdm. Our results confirmed that using phage display libraries was highly advantageous for the rapid development of molecules to detect target antigens. However, our results also indicated that this strategy might not have been effective for selecting H1N1pdm-specific antibodies during the 2009 pandemic, where the co-circulating sH1N1 virus shared similar antigenic properties. This suggests that it might be advisable to use a synthetic scFv phage display library by strategically considering the characteristics of target antigens and the potential situations.

## Findings

### Background

The periodic occurrence of influenza type A virus with novel antigenic properties, also known as a pandemic, has posed serious threats to the human population. Aiming to mitigate the disease burden posed by pandemics, various studies have investigated topics ranging from predictions of the potential subtypes that will cause the next pandemic [1] to the development of specific diagnostic tools and prepandemic vaccines [2]. These studies mainly targeted highly pathogenic avian influenza (HPAI) viruses typified by the H5N1 subtype [3]. In 2009, a novel influenza A/H1N1 virus (H1N1pdm)—a triple reassortant of “swine-origin” viruses [4]—emerged in Mexico and quickly spread across the globe. Co-circulation of H1N1pdm and then-existing seasonal H1N1 virus (sH1N1) created a need for distinguishing these viruses in clinical settings to better understand the pattern of transmission and pathogenesis of H1N1pdm [5]. Distinguishing between these viruses was also critical to choosing appropriate anti-influenza drugs, as the majority of sH1N1 in Japan, had acquired resistance against the orally active neuraminidase inhibitor oseltamivir by 2009 [6]. This situation prompted us to rapidly develop a point-of-care testing system during the brief time available before entering the 2009–2010 influenza season to differentiate H1N1pdm from sH1N1.

Immunochromatography (IC) has been routinely used in point-of-care rapid detection systems against seasonal influenza viruses. However, ICs that were commercially available at the onset of the 2009 pandemic, which targeted nucleoproteins (NPs), had been found not to distinguish between these 2 viruses. The development of IC designed to specifically detect H1N1pdm would be feasible if we could obtain an agent that specifically detected H1N1pdm but not sH1N1. Establishment of monoclonal antibodies was 1 option; however, this procedure requires animal immunization, which takes several months. We therefore decided to use phage display libraries to select H1N1pdm-specific single-chain variable fragments (scFvs). An scFv is an antigen-binding protein that consists of the V_H_ and V_L_ regions of the variable antigen-binding sites of immunoglobulin, connected by a short linker sequence. We anticipated that specific scFvs could be selected in a minimum of less than 3 weeks using scFv phage display libraries.

In this article, we will describe the H1N1pdm-reactive scFvs obtained and the unexpected observation that most scFvs altered their specificity and cross-reacted with sH1N1 after conversion from the phage-bound to soluble form. Relating these findings to the antigenic resemblance between H1N1pdm and sH1N1, we will discuss the importance of strategically selecting the methods used for developing diagnostic tools depending on the characteristics of the emerging virus, particularly under the time pressure posed by pandemics.

### Results

#### Initial screening of phage-bound scFvs against H1N1pdm

In this study, human single-fold scFv libraries I + J (Tomlinson I + J; a kind gift from MRC Centre for Protein Engineering, Cambridge, United Kingdom) underwent selection for the ability to bind H1N1pdm virus particles. After 3 rounds of panning, a total of 1152 individual *Escherichia coli* TG1 clones (576 clones each from Tomlinson I and J) were obtained and grown in 96-well U-bottom plates. From these *E. coli* TG1 clones, monoclonal phage were produced by adding KM13 helper phage to each well.

The binding of these monoclonal phage to immobilized H1N1pdm viral particles was subsequently investigated using enzyme-linked immunosorbent assay (ELISA). sH1N1 particles and proteins derived from the allantoic fluid of mock-infected eggs (AF proteins) were used as control antigens. Among the 1152 phage tested, 58 phage clones (31 and 27 clones from Tomlinson I and J, respectively) reacted with H1N1pdm specifically or preferentially in 2 independent ELISA trials (data not shown). All 58 of these clones were found to be unreactive with AF-protein wells, indicating that non-specific binding to AF proteins or skim milk (in blocking solution) was unlikely to occur in these clones. In order to select genetically independent phage clones, the phagemid vector pIT2 was isolated from the respective phage and the encoded scFv genes were sequenced as described elsewhere [7]. Consequently, these clones were found to comprise 42 genetically independent clones (15 and 27 clones from Tomlinson I and J, respectively).

#### Production of soluble scFvs and specificity confirmation

**Figure 1:**
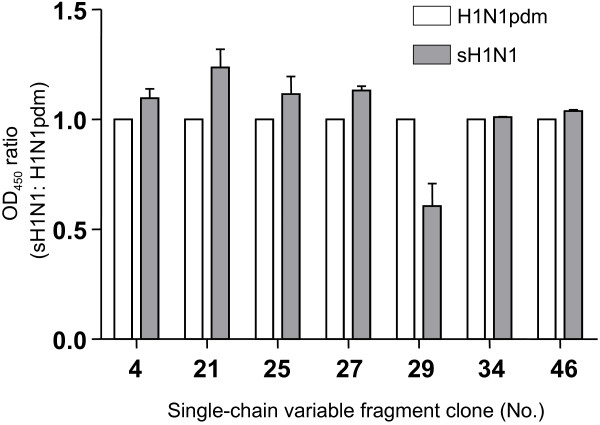
** Comparison of binding of respective soluble scFv against H1N1pdm and sH1N1 particles by ELISA.** To compare the binding ability of soluble scFv against H1N1pdm and sH1N1, ELISA was performed against each immobilized antigen in 2 independent trials. The ratio of OD_450_ against sH1N1 versus H1N1pdm is shown. Values are mean ± SE; N = 2.

**Figure 2:**
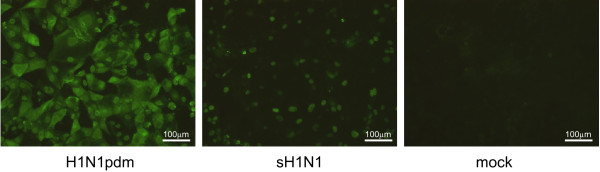
** Reactivity of soluble scFv No. 29 against H1N1pdm-, sH1N1-, and mock-infected MDCK cells in IFA.** IFA against H1N1pdm-, sH1N1-, and mock-infected MDCK cells was performed to examine the ability of soluble scFv No. 29 to bind virus. Following fixation of the cells using 3.6% formaldehyde and 0.4% Triton-X, 1.25 μg of scFv No. 29 was added and detected with an anti-myc tag MoAb and FITC-conjugated anti-mouse IgG.

Using these 42 phage clones, expression and purification of soluble scFvs was conducted. Expression levels of respective soluble scFv was confirmed by sodium dodecyl sulfate–polyacrylamide gel electrophoresis (SDS-PAGE), and we finally obtained 7 soluble clones that demonstrated high and stable expression in 2 independent expression trials (5 and 2 clones from Tomlinson I and J, respectively) (data not shown). These soluble scFvs were next subjected to ELISA against immobilized H1N1pdm and sH1N1. It was a surprise to find that 6 clones (Nos. 4, 21, 25, 27, 34, and 46) lost their specificity/preference toward H1N1pdm and showed equivalent reactivity against both viruses or even a slight preference toward sH1N1 (Figure 1). On the other hand, only 1 clone (No. 29 from Tomlinson I), maintained its preference toward H1N1pdm. In 2 independent ELISA trials, the same results were obtained. The reactivity of No. 29 against 2 viruses was also investigated using indirect immunofluorescence assay (IFA). While No. 29 showed a preference toward H1N1pdm (Figure 2), the other 6 clones reacted with both viruses equally (data not shown).

#### Further characterization of the soluble scFv clones

**Figure 3:**
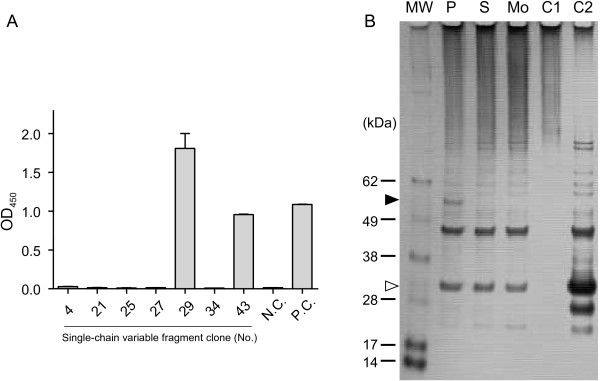
** A) Comparison of binding of respective soluble scFv against H1N1pdm and sH1N1 particles by ELISA.** To compare the binding ability of soluble scFv against purified H1N1pdm NP, ELISA was performed against each immobilized antigen in duplicate. N.C. represents negative control (soluble scFv against rabies virus phosphoprotein). P. C. indicates positive control (serum of H1N1pdm-infected ferret). Values are mean ± SE; N = 2. **B) Identification of the target protein of scFv No. 29 by immunoprecipitation and SDS-PAGE.** The lysates of H1N1pdm/sH1N1-infected and mock-infected MDCK cells were immunoprecipitated with soluble scFv No. 29 followed by SDS-PAGE analysis and silver staining. Lysate origins include H1N1pdm-infected cells (P), sH1N1-infected cells (S), and mock-infected cells (Mo). C1 and C2 indicate negative control reactions; only the lysate of mock-infected cells (C1) and the soluble scFv fluid (C2) were subjected to magnetic beads before following the same procedure as described for the other samples. ‘MW’ indicates a protein molecular weight marker. Filled arrow corresponds to the 56 kDa protein, presumably NP. Empty arrow denotes soluble scFvs. Other bands were considered to be non-specific binding of unrelated proteins.

To further characterize these 7 clones, with particular focus on No. 29, we attempted to identify viral proteins recognized by each scFv. First, hemagglutination inhibition (HI) and neutralization tests were performed to investigate whether these scFvs recognized envelope proteins. Neither HI nor neutralizing activity was observed (data not shown). Next, ELISA against purified H1N1pdm nucleoprotein (NP) was performed. Two clones, Nos. 29 and 46, were found to react with NP (Figure 3A). To confirm the reactivity of No. 29 against NP, immunoprecipitation was conducted against the lysates of MDCK cells infected with H1N1pdm or sH1N1 and mock-infected cells. As shown in Figure 3B, No. 29 reacted with a 56 kDa protein in the lysate of H1N1pdm-infected cells only. Based on the molecular weight, this protein was assumed to be NP.

### Discussion

In this study, scFvs against pandemic influenza type A virus from a phage display library were selected in order to generate an H1N1pdm-specific detection tool as quickly as possible before entering the first influenza season after emergence of the virus.

In the initial screening of phage-bound scFvs, we obtained a total of 58 clones that bound specifically to H1N1pdm or preferentially to H1N1pdm over sH1N1. After conversion to the soluble form, 7 genetically independent clones demonstrating high and stable expression were finally selected. Unexpectedly, 6 of the 7 soluble scFv clones were found to have lost their preference/specificity toward H1N1pdm (Figure 1). Interestingly, this change in specificity of scFvs after conversion from the phage-bound to soluble form has been previously described; Goswami *et al.*[8] reported the altered specificity of scFvs while attempting to isolate scFv specific to the placental isozyme of alkaline phosphatase (PLAP) using Tomlinson’s phage display library. Several clones that had been specific to PLAP in phage-bound form were observed to become cross-reactive with very closely related bone alkaline phosphatase isozymes after conversion to the soluble form. The authors speculated that this alteration in specificity was due to the loss of phage scaffold pIII protein associated with the production of soluble scFv. Because this protein has a role in anchoring scFv to the phage surface, they suggested that pIII might provide indirect structural support for the formation of the scFv antigen-binding site. Their observations provide a likely explanation for our results as well. A lack of structural support by pIII could account for the loss of scFv specificity against H1N1pdm in most clones after conversion from the phage-bound to soluble form. It is also possible that the similarity in antigenic properties shared by target proteins from identical subtypes of influenza A virus, H1N1pdm and sH1N1, could have led to the loss of scFv specificity with the high frequency observed in our study. A similar phenomenon was also reported recently in another study using Tomlinson’s phage display library. Chan *et al.*[9] observed the loss of specificity of scFvs against the protective antigen toxin component of *Bacillus anthracis* during conversion from phage-bound to soluble form. Considering several independent studies have produced comparable results using different antigens, there is a possibility that this poor convertibility of scFvs was not particular to the antigen used (such as influenza viruses) but inherent in this library.

Our strategy, panning on immobilized viruses, has been used in several previous studies [10,11] and contributed to the expedition of the whole selection process by eliminating the need to purify specific target antigens. We had originally expected that the use of purified viral particles as an immobilized antigen for panning would isolate scFvs against envelope proteins, which could overcome the inability of the then-used point-of-care ICs using anti-NP antibodies to differentiate H1N1pdm from sH1N1; NPs possess important antigenic differences that enable influenza viruses to be distinguished into 3 genera (influenza virus A, B, and C); however, NPs are highly conserved within each genus. On the other hand, HA genes are one of the most specific genes in the influenza virus genome, and viral RNA analysis targeting HA genes was conducted for specific detection of H1N1pdm in clinical specimens during the 2009 pandemic [12]. However, contrary to our expectations, among the 7 clones finally established, the target proteins of 2 scFv clones were identified as NP. Further investigation is needed to identify the target proteins of the other 5 clones. Although it remains unclear why anti-NP scFvs were selected, it was likely that some viral components had been exposed during the preparation of viral particles. In fact, during and after the 2009 pandemic, the development of several ICs, which can differentiate these 2 viruses were reported. These ICs target NP [13,14] or/and HA [13], and the NP-based system appeared to be superior to the HA-based system in terms of specificity and sensitivity [13]. This indicates that targeting NP, instead of envelope proteins, might have increased the possibility of obtaining H1N1pdm-specific scFvs in our study. However, considering the altered specificity of the scFvs observed in this study and in several previous studies [8,9], our unexpected results can be attributed to using a synthetic scFv phage display library rather than purified viral particles as an immobilized antigen for panning to target envelope proteins.

Since the 2009 pandemic, H1N1pdm has remained prevalent and is expected to replace sH1N1. Various studies have been conducted in anticipation of the next pandemic. Such research is important to prepare for the appearance of unexpected subtype strains that have the potential to become pandemic. As in the 2009 pandemic, such a situation would require the development of specific diagnostic tests or a vaccine in an extremely short period of time. Success would require that limited resources for research be allocated strategically, prudently, and according to feasibility. Our findings indicate that the use of a synthetic scFv phage display library might not necessarily be effective in every situation. Nevertheless, considering its remarkable rapidity in producing specific antibodies, it should be noted that this strategy could be highly advantageous in more suitable situations where a novel virus possesses distinct antigenic properties. Employing and combining appropriate strategies according to the characteristics of the emerging virus is essential for rapid development of diagnostic tools and mitigating the burden posed by a pandemic.

### Methods

#### Viruses and cells

Two strains of Influenza type A virus were used in this study: a 2009 pandemic strain, A/Narita/1/2009(H1N1) (referred to as “H1N1pdm”) and a seasonal influenza strain (2009), A/Brisbane/59/2007(H1N1) (referred to as “sH1N1”). These viruses were propagated in fertilized hens’ eggs. MDCK cells derived from canine kidney were used for investigating the interaction between scFvs and viral proteins by indirect fluorescent assay and immunoprecipitation (details follow).

#### Virus purification

Viral particles were purified from the AF of virus-infected fertilized hens’ eggs using Cellufine Sulfate (Chisso, Tokyo, Japan) following AF collection 48 h after inoculation. Briefly, after the Cellufine Sulfate gel was equilibrated with 0.01 M phosphate buffer (PB) (pH 7.4), the AF was mixed with the gel using a rotator for 1 h at room temperature. After washing 4 times in washing buffer (0.01 M PB, 0.19 M NaCl [pH 7.2]), viral particles were eluted with elution buffer (0.01 M PB, 3 M NaCl [pH 7.0]). The HA titer of each preparation was measured after every purification trial. As a negative control, AF from mock-infected eggs underwent the same procedure as above, and the eluted sample was referred to as “AF proteins”.

#### scFv phage display library

Human single-fold scFv libraries I + J (Tomlinson I + J; a kind gift from MRC Centre for Protein Engineering, Cambridge, United Kingdom) underwent selection for the ability to bind H1N1pdm viral particles. The libraries are based on a single human framework for V_H_ (V3-23/DP-47 and J_H_4b) and Vκ (O12/O2/DPK9 and Jκ1) with side chain diversity incorporated into positions in the antigen-binding site. The scFv genes in the libraries are linked to a His-tag followed by a myc tag and cloned into pIT2 phagemid vectors.

#### Selection of phage-bound scFvs

The selection or panning process was essentially as described in the manufacturer’s protocol [15]. Briefly, 3 Nunc immunotubes (Thermo Fisher, Wiesbaden, Germany) were coated overnight with 4 mL of a 1:10 dilution of the following antigens: i) H1N1pdm, ii) sH1N1, and iii) AF proteins. After washing with phosphate-buffered saline (PBS) and blocking with PBS containing 2% (w/v) skim milk, tubes were loaded with 10^12^ to 10^13^ phage in the first round of panning. In order to pre-exclude the phage reactive toward AF proteins or sH1N1, the phage were first applied to an AF-protein–coated tube and incubated for 2 h. Unbound phage were transferred to a sH1N1-coated tube, treated likewise, and finally reacted with an H1N1pdm-coated tube in the same manner. After intensive washes, bound phage were eluted by adding 500 μL of trypsin-PBS (2 mg/mL trypsin in PBS). The eluted phage were propagated and amplified in *E. coli* TG1. A total of 3 rounds of panning was conducted. For the second and third rounds of panning, only the H1N1pdm-coated tube was used.

#### ELISA

For initial screening of phage-bound scFvs, the ELISA plate was coated overnight with either H1N1pdm or sH1N1 or AF proteins. The amount of viruses coated were standardized to the same HA titer of 16 HA units per well. AF proteins were prepared by diluting AF of mock-infected eggs at the same dilution as the H1N1pdm preparation that had a lower HA titer. Non-specific binding was blocked using PBS containing 5% (w/v) skim milk. The phage (diluted 1:10) were added to a set of 3 wells coated with the respective antigen, and the ELISA plates were incubated for 1 h at room temperature. After the plates had been washed, the bound phage were visualized by the addition of horseradish peroxidase (HRP)-conjugated protein L (Pierce, Rockford, IL, USA) and SureBlue TMB 1-component Microwell Peroxidase Substrate (KPL, Gaithersburg, MD, USA). For the screening of soluble scFvs, the ELISA plate was coated with H1N1pdm and sH1N1. The soluble scFvs at a concentration of 8 μg/well were added to a set of 2 wells coated with the respective antigen, and the above procedure was followed. The optical density (OD) was read at 450 nm using a Model 680 Microplate Reader (Bio-Rad, Hercules, CA, USA). The cut-off OD_450_ was set at 0.6 against H1N1pdm. The OD ratio data was graphed using Prism 5 (GraphPad, San Diego, CA, USA). For the binding assay of soluble scFvs against H1N1pdm NP, 2 μg/well of purified NP (a kind gift from Dr. M. Tobiume, National Institute of Infectious Diseases) was coated onto the ELISA plate. Soluble scFvs at a concentration of 8 μg/well were added in duplicate, and the above procedure was followed. As a negative control, the same amount of a soluble scFv against rabies virus phosphoprotein [7] was used. As a positive control, 1:20 diluted H1N1pdm-infected ferret serum (a kind gift from Dr. T. Odagiri, National Institute of Infectious Diseases) was used.

#### Phagemid DNA sequencing

The phagemid vector pIT2 was isolated using a Qiagen Plasmid Midi Kit (Qiagen, Venlo, Netherlands). The phagemids were sequenced using the Big Dye Terminator v3.1 Cycle Sequencing Kit (Applied Biosystems, Foster City, CA, USA) with the 373 DNA Sequencer (Applied Biosystems). The primers used to sequence the scFv inserts in the pIT2 vector were LMB3 (5'-CAGGAAACAGCTATGAC-3′) and pHEN (5′-CTATGCGGCCCCATTCA-3′). Deduced amino acid sequences of each scFv were aligned using GENETYX Ver. 9 (Genetyx, Tokyo, Japan).

#### Production of soluble scFvs

Expression and purification of soluble scFvs were performed as described elsewhere [7]. Briefly, *E. coli* HB2151 cells were infected with these phage, and protein expression was induced by adding 1 mM isopropyl-β-d-thiogalactapyranoside to the culture. After purification of soluble scFvs using Ni-NTA nickel-charged resin (Qiagen, Venlo, Netherlands), SDS-PAGE was performed to confirm the expression of scFvs.

#### IFA

MDCK cells were infected with H1N1pdm and sH1N1 independently. After 48 h, the cells were fixed in 3.6% formaldehyde with/without 0.4% Triton-X. Ten or 1.25 μg of each soluble scFv was added and stained using anti-myc tag mouse MoAb (MBL, Nagoya, Japan) as the primary antibody and an FITC-goat anti-mouse IgG (H + L) (Invitrogen) as the secondary antibody. Cells were visualized, and the images were digitally captured using a BZ-8000 “Bio-zero” fluorescence microscope (KEYENCE, Osaka, Japan).

#### Immunoprecipitation

To identify the target protein of scFv No. 29, immunoprecipitation of soluble No. 29 against virus-infected MDCK cells was performed. Briefly, MDCK cells were grown as a monolayer in 6-well plates, infected with H1N1pdm or sH1N1 at a multiplicity of infection of 10, and subsequently incubated in a 5% CO_2_ incubator. After 48 h, the cells were lysed with 0.5 mL of lysis buffer (50 mM sodium phosphate [pH 8.0], 300 mM NaCl, 0.01% [vol/vol] Tween-20, 1% [vol/vol] Triton-X 100) at 4°C for 1 h. The cell lysates were centrifuged at 20 000 × *g* for 30 min at 4°C. The supernatants were collected and reacted with 2.5 μg of scFvs for 1 h at 4°C. The antigen-scFv complexes were immunoprecipitated using magnetic beads (Dynabeads TALON; Invitrogen, Carlsbad, CA, USA) according to manufacturer’s protocol. The eluted samples were subjected to SDS-PAGE and silver stained using EzStain Sliver (ATTO, Tokyo, Japan) according to manufacturer’s protocol.

## Competing interests

The authors declare that they have no competing interests.

## Authors’ contributions

YK, SI and AY designed the framework of the study. YK, AN, KT, YY, AH, MS and NS conducted virus propagation and purification. YK, AN, KT and YY performed the screening of phage-bound and soluble scFvs. AN and AO conducted genetic analysis of the selected phagemids.YK drafted the original manuscript. All authors have participated in the final editing and approved the final manuscript.
